# Carvacrol attenuates serum levels of total protein, phospholipase A2 and histamine in asthmatic guinea pig 

**Published:** 2016

**Authors:** Mohammad Hossein Boskabady, Sedigheh Jalali, Negin Yahyazadeh, Mostafa Boskabady

**Affiliations:** 1 *Neurogenic Inflammation Research Centre and Department of Physiology, School of Medicine, Mashhad University of Medical Sciences, Mashhad, Iran*; 2 *Department of Biology, Payam Noor University, 19395-4697 Tehran, Iran*

**Keywords:** *Carvacrol*, *Guinea pigs*, *Asthma*, *Sensitization*, *Total protein*, *Phospholipase A2*, *Histamine*

## Abstract

**Objective:**

Pharmacological effects of carvacrol such as its anti-inflammatory activities have been shows. In this study the effects of carvacrol on serum levels of total protein (TP), phospholipase A2 (PLA2) and histamine in sensitized guinea pigs was evaluated.

**Materials and Methods:**

Sensitized guinea pigs were given drinking water alone (group S), drinking water containing three concentrations of carvacrol (40, 80 and 160 µg/ml) or dexamethasone. Serum levels of TP, PLA2 and histamine were examined I all sensitized groups as well as a non-sensitized control group (n=6 for each group).

**Results:**

In sensitized animals, serum levels of TP, PLA2 and histamine were significantly increased compared to control animals (p*<*0.05 to p*<*0.001). Significant reduction in TP, PLA2 and histamine levels were observed in treated groups with the two higher concentrations of carvacrol but dexamethasone treatment only decreased serum level of PLA2 (p<0.05 to p*<*0.001). Although the effect of the lowest concentration of the extract was less than that of dexamethasone (p*<*0.05 for TP and p*<*0.001 for PLA2), the effects of the two higher concentrations on PLA2 were similar to dexamethasone and on TP (p*<*0.01) and histamine (p*<*0.001) were higher than those of dexamethasone.

**Conclusion:**

These results showed that carvacrol reduced serum levels of TP, PLA2 and histamine in sensitized guinea pigs which may indicate an anti-inflammatory effect of this agent in inflammatory disorders such as asthma.

## Introduction

Carvacrol (cymophenol, C_6_H_3_CH_3_ (OH) (C_3_H_7_)) is one of the main constituent of *Zataria multiflora *Bois (*Z. multiflora*), (ESCOP, 1997 ▶). Therapeutic effects *Z. multiflora* and its constituent, carvacrol on common colds, bronchitis, pertussis, laryngitis, and antibacterial activity in oral hygiene and stomatitis have been described (ESCOP, 1997 ▶). The relaxant effect of plant containing carvacrol on different smooth muscles including trachea (Boskabady et al., 1998 ▶; Boskabady et al., 2006 ▶, Boskabady et al., 2007 ▶) has been previously shown. as well as Carvacrol (Boskabady and Jandaghi, 2003 ▶) and fraction 2 of the essential oil from *Carum copticum *which is suggested to be carvacrol (Boskabady et al., 2003 ▶) showed relaxant effect on tracheal smooth muscle. Various pharmacological effects including antibacterial (Nostro et al., 2007 ▶) and anti-candidiasis effects (Knowles et al., 2005 ▶), the effect on DNA binding (Nafisi et al., 2004 ▶), genotoxicity (Zeytinoglu and Baser, 2003 ▶), antimutagenicity (Ipek et al., 2005 ▶), antigenotoxic (Ipek et al., 2003 ▶), anti-cancer (Mehdi et al., 2011 ▶) and apoptosis inducing (Akalin and Incesu, 2011 ▶), anti-inflammatory and anti-oxidant (Hotta et al,. 2010 ▶; Landa et al., 2009 ▶) properties were shown.

The main characteristic of asthma is airway inflammation is (Busse et al., 1995 ▶) which different inflammatory cells are activated in this process (Kelly et al., 1998 ▶). Phospholipase A2 (PLA2) is released from inflammatory cells activated due to airway inflammation in asthma (Vadas and Pruzanski, 1986 ▶). PLA2, in turn, leads to synthesis of eicosanoids by various inflammatory cells (Vadas and Pruzanski, 1986 ▶). In the allergic inflammation of bronchial asthma, eicosanoids play an important role (Kashima et al., 1993 ▶). In serum and bronchoalveolar lavage fluid from asthmatic patients, increased PLA2 activity was shown (Kashima et al., 1993 ▶). In addition, increased serum histamine level in asthmatic patients and animals, was also documented (Busse and Swenson, 1989 ▶). Increased serum total protein was also shown in subjects with occupational asthma (Qureshi et al., 2009 ▶).

The treatment for asthma consists of two types of drugs: 1) bronchodilatory drugs to relieve bronchoconstriction and 2) anti-inflammatory or preventive drugs to suppress the airway inflammation. With regard to relaxant and anti-inflammatory effects of carvacrol, this agent may affect asthma therapy by both mechanisms. In the present study, the effect of carvacrol on histamine, PLA2 and TP in sensitized guinea pigs was examined.

## Materials and Methods

**Animal groups**

Control guinea pigs (group C, treated the same as sensitized group but normal saline was used instead of OA and they were given drinking water alone) and five groups of sensitized animals were which given drinking containing the following agents (n=6 for each group) were studied.

1) Drinking water alone (group S, an animal model of asthma)

2) µg/ml dexamethasone (group S+D)

3) 40 µg/ml carvacrol (group S+C1)

4) µg/ml carvacrol (group S+C2)

5) 160 µg/ml carvacrol (group S+C3)

Sensitization of guinea pigs with OA as was performed as previously described (Boskabady and Ziaei , 2003 ▶; Jafari et al., 2011 ▶; Vosooghi et al., 2013 ▶). Briefly, guinea pigs were sensitized with 10 mg of OA (Sigma Chemical Ltd, UK) + 100 mg of Al(OH)_3_ dissolved in 1 ml saline on first day and 2 mg of OA + 100 mg of Al(OH)_3_ dissolved in 1 ml saline i.p. one week later. From day 14, sensitized animals were exposed to an aerosol of 4% OA for 18±1 days, for 5 min daily. The aerosol was administered in an enclosed chamber (30 x 20 x 20 cm). Control animals were treated similarly but saline was used instead of OA solution. The study was approved by the ethical committee of Mashhad University of Medical Sciences, Mashhad, Iran.

**Measurement of serum TP, PLA2 and histamine**

Animals were sacrificed after the end of sensitization period (32±1 days). Five milliliter peripheral blood was obtained immediately after sacrificing of animals. Blood samples were kept at room temperature for 1 hr. The samples were then centrifuged at 2500×g at 4 °C for 10 min. The supernatant was collected and stored at -70 °C until analyzed. The serum protein level was determined using quantitative protein assay kit (Pars Azmoon, Iran) according to the manufacturer’s protocol with photometric method. Using the Enzyme-Linked Immuno-Sorbent Assay (ELISA) sandwich method, serum PLA2 and histamine levels were measured according to the manufacturer’s instructions (PLA_2_: E10217: Invitrogen, England LDN’s Histamine Assay Kit, BAE-1100, Co., LDN, England). 

**Statistical analysis**

Results were expressed as mean±SEM. Kolmogorov-Smirnov test indicate the normal distribution of the data. Comparison between the results of sensitized group and control guinea pigs as well as between sensitized and treated groups were performed using unpaired "t" test. Data of three groups of animals treated with carvacrol were compared using one way ANOVA with Tukey-Kramer *post-hoc* test. Significance was accepted at p<0.05.

## Results

**Figure1:**
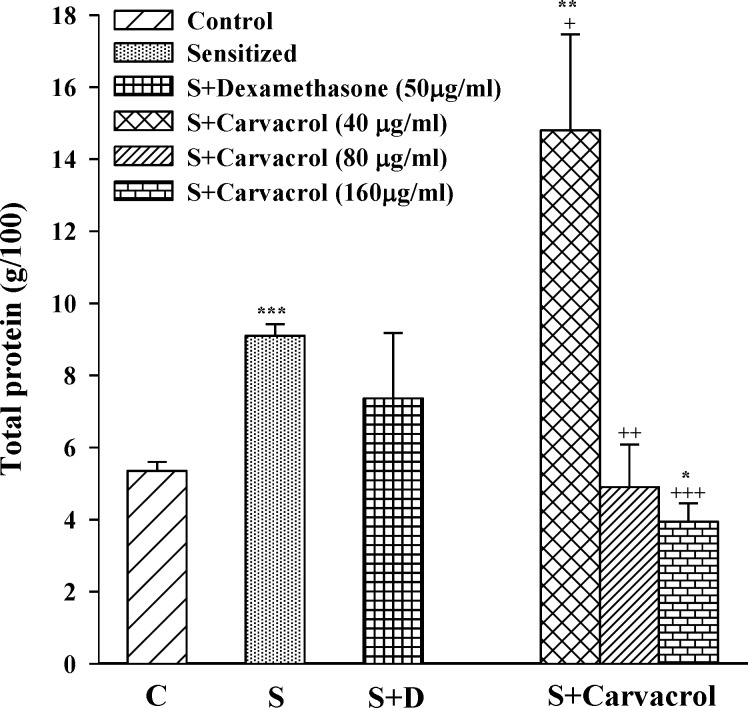
Serum levels of total protein in control group (C), sensitized guinea pigs (S), S treated with dexamethasone and three concentrations of carvacrol (n=6). Statistical differences between control and other groups: *p<0.05, **p<0.01 and ***p<0.001. Statistical differences between treated animals *vs *sensitized group: +p<0.05, ++p<0.01 and +++p<0.001

**Figure 2:**
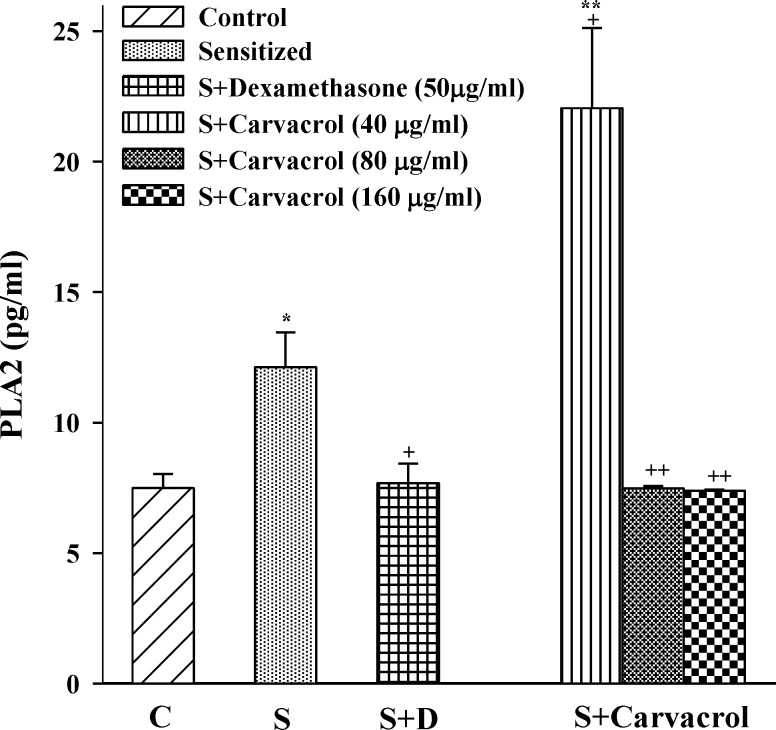
Serum levels of phospholipase A2 in control group (C), sensitized guinea pigs (S), S treated with dexamethasone and three concentrations of carvacrol (n=6). Statistical differences between control and other groups: *p<0.05 and **p<0.01. Statistical differences between treated animals *vs *sensitized group: +: p<0.05 and ++: p<0.01

**Figure 3:**
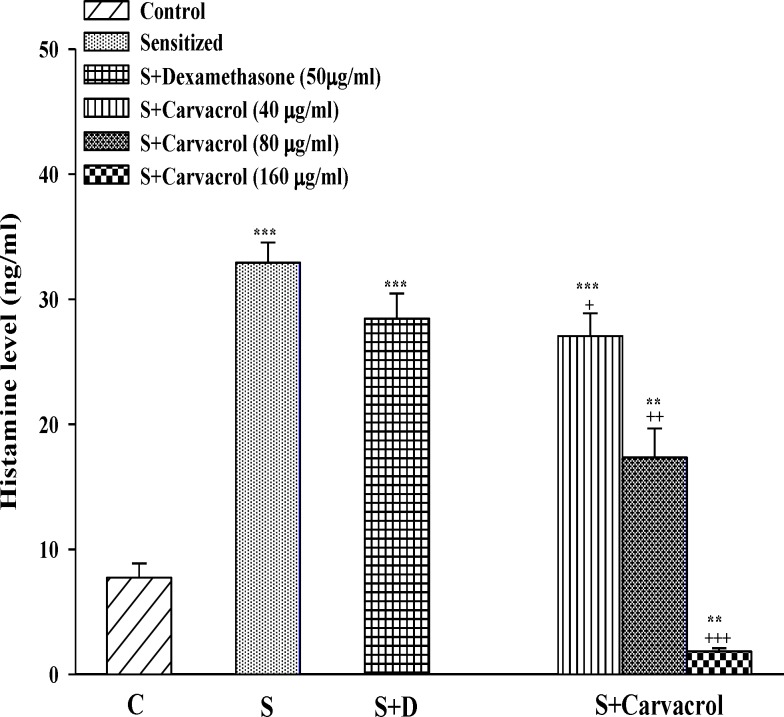
Serum levels of histamine in control group (C), sensitized guinea pigs (S), S treated with dexamethasone and three concentrations of carvacrol (n=6). Statistical differences between control and other groups: **p<0.01 and ***p<0.001. Statistical differences between treated animals *vs *sensitized group: ++p<0.01, +++p<0.001 and +++p<0.001

In sensitized group, serum levels of TP, PLA2 and histamine were significantly higher than control animals (p<0.05 for PLA2 and p<0.001 for TP and histamine ) (Figure 1-3). Serum levels of TP, PLA2 and histamine were significantly decreased in treated groups with the two higher concentrations of carvacrol (p<0.01 to p<0.001, Figure 1-3). 

However, only serum level of PLA2 was significantly decreased in S animals treated with dexamethasone (p<0.05) (Figure 2).

The effect of the lowest concentration of carvacrol (40 µg/ml) on TP and PLA2 was significantly less than the effect of dexamethasone (p<0.05 for TP and p<0.001 for PLA2; Table 1). However, the effect of the highest concentrations of carvacrol (160 µg/ ml) on PLA2 was similar to dexamethasone and on TP (p<0.01) and histamine (p<0.001) was higher than that of dexamethasone (Table 1). The effects of the two higher concentrations of carvacrol (80 and 160 µg/ ml) on serum levels of TP, PLA2 and histamine were significantly higher (p< 0.001 for histamine and p< 0.01 for other parameters) than the effect of the lowest concentration (Table 1). In addition, the effect of the highest concentration of carvacrol on histamine level was also significantly higher than the effect of its medium concentration (p<0.001, Table 1).

**Table 1:** Serum levels of total protein, PLA2 and histamine levels in control group, sensitized guinea pigs (s), s treated with three concentrations of carvacrol (S+C1, S+C2, S+C3) and dexamethasone (S+D), (n= 6).

**Mediator**	**Control**	**S**	**S+D**	**S** **+C1**	**S+C2**	**S+C3**
**Total protein**	5.35±0.25	9.1±0.32	7.36±1.81	14.80±2.66	4.90±1.18	3.94±0.51
**PLA2**	7.49±0.54	12.12±1.34	7.68±0.75	22.05±3.07	7.48±0.09	7.39±0.04
**Histamine**	7.73±1.15	32.92±1.62	28.45±2.01	27.05±1.81	17.34±2.3	1.84±0.25***

Values are presented as mean±SEM. Three concentrations of carvacrol were 40 (S+C1), 80 (S+C2) and 160 µg/ml (S+C3) and dexamethasone was used at the concentration of 50 μg/ml. Statistical *significance* for the *difference* between the data of S+C3 and S+C1 vs S+C1:

p<0.01,

p<0.001. Statistical *significance* for the *difference* between the data of S+C3 vs S+C2:

*** p<0.001. The statistical comparisons were made using ANOVA with Tukey- Kramer multiple post test.

## Discussion

Increased serum levels of TP, PLA2 and histamine was showed in sensitized guinea pigs in this study. Previous studies also showed increased serum levels of total protein (Qureshi et al., 2009 ▶), PLA2 (Vadas and Pruzanski, 1986 ▶) and hitamine (Busse and Swenson, 1989 ▶) in asthmatic patients and animals which confirm the sensitization (induction of animal model of asthma) in the present study. 

The two higher concentrations of carvacrol led to reduction in serum levels of TP, PLA2 and histamine in sensitized animals. However, dexamethasone treatment did not affect serum level of TP and histamine and only caused significant reduction in PLA2 level. Airway inflammation is the most important feature of asthma (Busse et al., 1995 ▶). Therefore, in asthma-therapy we should focus on reducing airway inflammation.

The results of the present study showed that carvacrol prevents the increase in serum levels of TP, PLA2 and histamine in sensitized guinea pigs. However, dexamethasone treatment only affects serum level of PLA2 in sensitized guinea pigs. These results indicate that carvacrol have a therapeutic effect on inflammatory markers in sensitized animals which is more potent, more specific and thus different from the effect of dexamethasone. Therefore, the results of the present study showed that in inflammatory disorders such as asthma, carvacrol may have even more therapeutic values compared to dexamethasone. In fact, anti-inflammatory and anti-oxidant effects of carvacrol were previously shown (Hotta et al., 2010 ▶; Landa et al., 2009 ▶) which are consistent with the results of the present study.

The results also showed that the effect of carvacrol on serum levels of TP, PLA2 and histamine was almost concentration-dependent. The greater effect of higher concentrations of carvacrol on serum levels of TP and histamine compared to dexamethasone suggests that the effect of carvacrol is greater than dexamethasone on most inflammatory markers in sensitized guinea pigs at used concentrations.

A potent relaxant effect of the extract of a plant containing carvacrol (*Thymus vulgaris*) (Boskabady et al., 2006 ▶) and carvacrol itself (Boskabady and Jandaghi., 2003 ▶) on tracheal chains, stimulatory effect of *Z. multiflora* (another plant containing carvacrol) and carvacrol on ß_2_-adrnoceptors (Boskabady et al., 2011a ▶) and their inhibitory effect on histamine (H1) receptors (Boskabady et al., 2012a ▶) were previously shown. The inhibitory effect of carvacrol (Boskabady et al., 2011b) and the extract of *Z. multiflora *(Boskabady et al., 2012b ▶; Jafari et al., 2011 ▶) on muscarinic receptors of tracheal smooth muscle were also demonstrated. The present study also showed preventive effect on inflammatory markers in sensitized animals. Therefore, the results of the present study as well as previous studies suggest that carvacrol may have therapeutic effect on asthma by both bronchodilation as a relieving drug and anti–inflammatory effect as a preventive drug. The effects of other natural products on lung inflammation and tracheal responsiveness were shown using similar methods which support the findings of the present study (Boskabady and Ziaei, 2003 ▶; Vosooghi et al., 2013 ▶). However, more studies on the effect of carvacrol on asthmatic patients are needed to confirm this suggestion.

In conclusion, reduction effect of carvacrol on serum levels of TP, PLA2 and histamine in sensitized guinea pigs we indicated. The effect of carvacrol was more specific compared to dexamethasone because dexamethasone treatment only affected PLA2 level in sensitized animals. Therefore, the results suggest that the effect of carvacrol is greater than dexamethasone on inflammatory markers in sensitized guinea pigs at used concentrations.
